# 

**Published:** 1852-04-01

**Authors:** 


Reviews of Sir James Eyre's interesting little volume, *The Stomach and its Difficulties*; Colquhoun's *Magic Mesmerism*, &c.; Dr. Dickson *On the Establishment of Public Hospitals for the Insane of the Middle and Higher Classes;* Dr. Cumming's *Notes of Lunatic Asylums in Germany;* reports of several County Asylums, with numerous other notices of works and pamphlets, are unavoidably postponed until our next number, in order to enable us to publish a full report of the inquiry into the alleged Lunacy of Mrs. Cumming. We have also been compelled to lay aside, for the present, several important medico-legal investigations involving questions of Insanity, and an elaborate report of *Legal Cases in Insanity*, argued before the Lord Chancellor and the Lords Justices, prepared expressly for publication in this Journal. We would again intimate to our American correspondents, that we have been compelled, in consequence of the amount of postage demanded, to refuse several pamphlets forwarded to us from the United States. The last number of the *American Journal of Insanity* has not reached us. Several correspondents have addressed us in relation to Dr. H. Monro's suggestions for improving the condition of private asylums. We have no hesitation in admitting that our views are at variance with those promulgated by the author of the papers referred to. In our next number, we shall have an opportunity of stating, in detail, our objections.

---

### DR. WINSLOW'S LETTSOMIAN LECTURES,

(1) On the Psychological Character of the Physician,

(2) On the Medical Treatment of Insanity,

(3) On Medico-Legal Evidence in Cases of Insanity

Will be delivered at the Rooms of the Medical Society of London, George-street, Hanover-square, on the 7th, 14th, and 21st of April.

LONDON:
SAVILL AND EDWARDS, PRINTERS, CHANDOS STREET,
COVENT GARDEN.

